# COVID-19 Pandemic: Experiences of People with Visual Impairment

**DOI:** 10.17533/udea.iee.v39n1e09

**Published:** 2021-03-05

**Authors:** María del Pilar Oviedo-Cáceres, Karen Natalia Arias-Pineda, María del Rosario Yepes-Camacho, Patricia Montoya Falla

**Affiliations:** 1 Optometrist, Ph.D(c). Professor, Universidad Santo Tomás, Bucaramanga (Colombia). Email: maria.oviedo@ustabuca.edu.co Universidad Santo Tomás Universidad Santo Tomás Bucaramanga Colombia maria.oviedo@ustabuca.edu.co; 2 Social Communicator, Master’s. Professor, Universidad Santo Tomás, Bucaramanga (Colombia). Email: karen.arias01@ustabuca.edu.co Universidad Santo Tomás Universidad Santo Tomás Bucaramanga Colombia karen.arias01@ustabuca.edu.co; 3 Occupational Therapist. Grupo Gestión Interinstitucional-Investigación, Instituto Nacional para Ciegos-INCI, Bogotá (Colombia). Email: myepes@inci.gov.co Instituto Nacional para Ciegos-INCI Bogotá Colombia myepes@inci.gov.co; 4 Psychologist. Grupo Gestión Interinstitucional-Investigación, Instituto Nacional para Ciegos-INCI, Bogotá (Colombia). Email: pmontoya@inci.gov.co Instituto Nacional para Ciegos-INCI Bogotá Colombia pmontoya@inci.gov.co

**Keywords:** pandemics, Coronavirus infections, disabled persons, visually impaired persons, Internet, pandemias, infecciones por Coronavirus, personas con discapacidad, personas con daño visual, Internet, pandemias, infecções por Coronavirus, pessoas com deficiência, pessoas com deficiência visual, Internet

## Abstract

**Objective.:**

To understand changes in daily life emerging from the COVID-19 Pandemic in people with visual impairment from four cities of Colombia.

**Methods.:**

Exploratory-type, descriptive qualitative study. The study conducted 26 semi-structured interviews via telephone. The analysis process used the methodological design from the approach proposed by Taylor and Bogdan: following the discovery process, coding and relativizing of data.

**Results.:**

Three categories emerge: 1) Transformations in daily dynamics, 2) Barriers to mobility, and 3) Use of technology.

**Conclusion.:**

People with visual impairment report barriers to mobility to take public transportation, which can affect maintenance of their autonomy and independence. Using technological tools is identified as facilitators for the continuity of educational and work activities; however, some did not have computer literacy or the basic inputs for connectivity. Difficulties were identified to continue work activities and maintain income.

## Resumen

**Objetivo.:**

Comprender los cambios en la vida cotidiana de las personas con discapacidad visual de cuatro ciudades de Colombia debido a la pandemia del COVID-19.

**Métodos.:**

Estudio cualitativo descriptivo de tipo exploratorio. Se realizaron 26 entrevistas semi-estructuradas vía telefónica. Para el proceso de análisis, se utilizó el diseño metodológico desde el enfoque propuesto por Taylor y Bogdan, siguiendo el proceso de descubrimiento, codificación y relativización de los datos.

**Resultados.:**

Emergen tres categorías: 1) transformaciones en las dinámicas cotidianas; 2) barreras para la movilidad y 3) el uso de tecnología.

**Conclusión.:**

Las personas con discapacidad visual manifiestan barreras para la movilidad y la toma del transporte público, lo cual afecta su autonomía e independencia. El uso de herramientas tecnológicas facilita la continuidad de actividades educativas y laborales, sin embargo, algunos no contaban con la alfabetización digital o con los insumos básicos para la conexión. Se identifican dificultades para la continuación de actividades laborales y el mantenimiento de ingresos.

## Resumo

**Objetivo.:**

Compreender as mudanças na vida diária decorrentes da pandemia COVID-19 em pessoas com deficiência visual em quatro cidades da Colômbia.

**Métodos.:**

Estudo exploratório descritivo qualitativo. 26 entrevistas semiestruturadas foram realizadas por telefone. Para o processo de análise, o desenho metodológico foi utilizado a partir da abordagem proposta por Taylor e Bogdan: acompanhando o processo de descoberta, codificação e relativização dos dados.

**Resultados.:**

Surgem três categorias: 1) Transformações na dinâmica diária, 2) Barreiras à mobilidade e 3) Uso de tecnologia.

**Conclusão.:**

Pessoas com deficiência visual manifestam barreiras à mobilidade, para o uso do transporte público, que podem afetar a manutenção de sua autonomia e independência. A utilização de ferramentas tecnológicas é identificada como facilitadora para a continuidade das atividades educacionais e de trabalho; no entanto, alguns não tinham alfabetização digital ou as entradas básicas para conexão. São identificadas dificuldades para a continuidade das atividades laborais e a manutenção da sua renda.

## Introduction

The World Health Organization (WHO) estimates that 15% of the global population has a disability, that is, nearly 1-billion people. (1) In 2015 it was reported that close to 441.5-million people suffered visual impairment of which 36-million report blindness, 217-million had moderate to severe visual impairment (low vision), and another 188-million endured slight visual impairment. (2) In turn, it is calculated that the number of people with visual impairment could be triplicated by 2050, when there could be 115-million people. (3) Preliminary reports from the 2018 population census indicate a national prevalence of disability of 7.1% (3 065 361 people of which 1 784 372 report difficulties with levels of greater severity: serious or total disability); and type of difficulties, like low near or distance vision or around constitute 18.7%. According to these figures, visual impairment could occupy the second place in frequency among the population with disability in Colombia. (4)

In relation with the structural conditions, it is reported that visual impairment (includes low vision and blindness) affects disproportionately the vulnerable populations, given that low-income countries have higher prevalence than those with high income, found most commonly in women and older individuals in condition of poverty. In this sense, it is found that 90% of the global burden of visual impairment is concentrated in developing countries. (5) Hence, it is felt that poverty and visual impairment are cyclically linked, given that poverty increases the risk of visual impairment and such exacerbates poverty by limiting opportunities of participating in wage-earning activities. (6) For the specific case of visual impairment and bearing in mind the Location and Characterization Record of people with disability, it is identified that they are concentrated in socioeconomic levels one and two, which evidences their reduced opportunity of mobilization in the social structure and, in turn, only 5% of the people with disability reached the secondary level of education, suggesting poor opportunity of formation and with it a tight range of labor qualification. (7)

This is the context of exclusion and vulnerability lived by people with visual impairment, which has become even more complex since March 2020, when the WHO declared a global pandemic due to the COVID-19 outbreak, given that it is likely that the pandemic affects more those with disabilities because it has been reported that those people are more prone to being infected with SARS-Cov 2 and experience disproportionate effects of the confinement. (8) In this sense, the effects of the pandemic could have serious consequences on the health, wellbeing, and quality of life of people with visual impairment. According to the Center for Systems Science and Engineering at Johns Hopkins University, vulnerable people among which are included people with disability will be the most affected, besides having the highest probability of suffering devastating losses. (9)

For the specific case of Colombia, on 06 March 2020, the first case was confirmed, which was imported from Italy. Upon the drafting of this this text -28 October 2020- the country already had 1,033,218 cases and 30,565 deaths. The pandemic, followed by confinement throughout the country to stop the unprecedented propagation of the virus, will have a serious impact on people with visual impairment. Many restrictive and control measures, including the adoption of new behavioral changes (for example, social distancing during movement out in the open, limited contact or tactile contact) recommended by the World Health Organization, pose huge challenges for people with visual loss. (10) The health situation, added to the sudden change in routines and roles that comprise our identity and which grant sense to our daily doings, may have had consequences at physical, social, psychological, and - above all - emotional levels, independent of the moment of the current vital cycle. (11) However, to date, no information exists on the impact caused by COVID-19 upon this group in particular or on their vulnerability. (9) In this sense, this study sought to understand the changes in daily life arising from the pandemic due to COVID-19 in people with visual impairment from four cities of Colombia: Bogotá, Arauca, Bucaramanga, and Piedecuesta.

## Methods

An exploratory-type, descriptive qualitative study was conducted to understand changes in daily life arising from the COVID-19 pandemic in people with visual impairment from four cities of Colombia: Bogotá, Bucaramanga, Piedecuesta and Arauca. Participant selection was intentional, using the special interview criterion, defined as those in a unique position in the community; to be considered in the research, the subjects had to be consenting adults and have low vision or blindness and reside in these geographic zones of the country, places prioritized by the National Institute for the Blind (*Instituto Nacional para Ciegos*) for accompaniment processes to organizations of people with visual impairment. A revision was carried out of the National Institute for the Blind database of people and organizations existing in said cities and an open invitation was made to participate via e-mail or mobile text messaging. People who manifested interest in participating were later contacted via telephone by two researchers. Each participant was explained the research objectives, methodology used, and the results expected from the study. They were requested a verbal informed consent to conduct the semi-structured interviews.

At all times, the study guaranteed ethical, anonymous, and confidential management of the information and names of the participants. This research was approved by the ethics committee at Universidad Santo Tomás, Bucaramanga branch, and adhered to that established in Resolution 008430 of 1993 by the Colombian Ministry of Health for norms in health research**.** Given the condition of isolation due to the pandemic, the tool used was the collection of information through a telephone semi-structured interview, which permitted approaching the experiences of people with visual impairment within the framework of the health emergency due to coronavirus. Prior to conducting the interviews, three group meetings were held in the Team platform to socialize with the individuals the research objectives and the process proposed, this permitted clearing doubts and uncertainties from the participants. Thereafter, the four researchers with experience in qualitative research and work with people with visual impairment interviewed each participant. Each telephone interview lasted an average of one hour. During the interviews, two researchers were always present; while one guided the interview, the other one took notes and included key aspects for analysis. All the interviews were magnetically recorded through a telephone application and were later identified by considering the city and assigning each of them an identification code. The interviews posed questions, like: how have the days of confinement been; how has confinement affected your daily routines; and which aspects have been critical or - on the contrary - positive?

The analysis process used the methodological design from the approach by Taylor and Bogdan, who proposed a method to analyze information from the findings given in the interviews; hence, the study followed the discovery process, coding, and relativizing of data. (12) The discovery phase sought emerging themes by examining the data, repeatedly reading, elaborating typologies, and developing possible concepts. Then, the coding phase analyzed all the data referring to themes, ideas, concepts, interpretations, and propositions. Finally, the relativizing stage interpreted the information in the context. To support the analysis of the reports by the participants, the Atlas ti (v. 6.2) software was used. The research had 26 participants; 10 from the city of Bogotá, 3 from Bucaramanga, 5 from Piedecuesta, and 8 from Arauca. The mean age was 40 years, with a minimum age of 21 years and a maximum of 68 years. Of all the participants, 53.9% were males.

## Results

Social isolation produced by the COVID-19 Pandemic has caused changes in the individual and family dynamics of the people interviewed. From the analysis of the results, three categories emerge: 1) Transformations in daily dynamics, 2) Barriers for mobility, and 3) Use of technology.

### Transformations in daily dynamics

For some, it has been an opportunity to come close to family members and strengthen bonds among their support networks: *For me, these days of pandemic have been about learning, finding myself, and a new encounter with the family; it has been a very pleasant thing, we are in a very beautiful town and have a spectacular landscape and every day my wife would describe the sunsets that she found phenomenal and we had not done this exercise before because it demanded a lot of work [E1. Piedecuesta. Man]. This is an opportunity to open the mind to new opportunities that surely will come up in the future [E2. Bogotá. Woman].*

The pandemic has meant a rupture for everyone in relation with the development of their daily activities and, of course, people with visual impairment have also experienced it. Although the situation has affected in generalized manner the global population, its effects are experienced in differentiated form in the distinct population groups; given that this situation has further marked the gaps of inequality and inequities lived by some of the people with visual impairment in the country, which impede coverage of basic needs: *Unemployment and poverty are tenacious; now in the pandemic a food crisis is being experienced, we have sounded all the alarms, minimum aid is arriving and people normally - when they can - eat every day, so their lives are very hard [E6. Bogotá. Woman].*

Before the pandemic, some of the people interviewed were informally employed or through temporary contracts, which were interrupted by closings in different sectors: *I was working as a commercial consultant in a foundation on themes of inclusion, but due to what is happening, the contract was suspended [E3. Piedecuesta. Man]. I had not started to work, I was going to start just when the quarantine began and the contract was suspended because, obviously, we did not know what was going to happen [E2. Bucaramanga. Man].*

The reports by the people interviewed account for the labor fragility or for the type of employment to which people with visual impairment can have access, which implies the need to strengthen labor inclusion processes in this population group: *Because of the pandemic I had to give up the garden, I left it aside and for the moment and I had to come to my parent’s house due to the situation, I am working independently in commercial sales from seven in the morning until eight at night [E1. Arauca. Woman]. I go to this lady and sometimes I help her with the cleaning in the miscellaneous store and she gives me some money, but that was before all this started. Sometimes I work in sales through catalogues, but with this quarantine I have not worked much in that because I have not been able to go out much. [E4. Bogotá. Woman]*

### Barriers for mobility

Barriers for mobility intensified due to COVID-19; a critical case has to do with the use of public transportation, given that this activity became an issue of greater difficulty for people with visual impairment. The norms related with social distancing, avoiding touching surfaces due to the risk, and not finding easily people willing to help in the street were aspects those interviewed identified as the biggest challenges, given that these are extremely difficult to comply, which leads people to feeling anxious and nervous: *You get scared, I say I can go out with all the protection and all, but fear one way or another affects because you will have much more contact with surfaces, the issue of taking the train, I have not taken the train again, since March I have not used mass transportation [E1. Piedecuesta. Man]. To use transportation require someone to help you, but people are distanced and many ignore you. I have had to scream to ask for a favor, but that is very difficult because people do not help. Because we are in a society in which we fear others, it does not help for people to understand that we are people who need support. They say there are protocols that people with disability have priority, that is not true, that is not complied [E2. Bogotá. Woman]*

This aspect has impacted negatively on autonomy and independence to carry out activities, given that most depend on touch to carry out their routine activities or movements out in the open, which can further increase the possibility of being infected by the virus: *Sometimes I have to seek other people, my neighbors, or my other sister to help me with certain things that sometimes I could go alone, but when you cannot go someplace alone, well you just can’t, so you have to take a companion [E8. Bogotá. Woman]. Before, you could go out alone without having to depend on another person and this time one way or another I had to rely on the companion to run errands [E3. Piedecuesta. Man].*

Added to the aforementioned, people with visual impairment have identified how during the management of the pandemic they have not been recognized as subjects with rights, with capacity for self-management and to care for their own health, which accounts for the negative imaginaries that persist in Colombian society: *Someone stated that we should be special beings of care, when we are people with disability, but it does not imply we have to be extremely cared for and protected like objects. We are not objects, we are humans who need to learn and put into practice the recommendations to safeguard life. [E5. Piedecuesta. Man]. I don’t like to be treated in special manner because I am not special [E3. Bucaramanga. Man].* This matter evidences the need to work to, especially in communication media and in community scenarios, avoid reinforcing negative stereotypes associated with visual impairment.

### Use of technology

Use of technology has been a fundamental element for the continuity of educational, labor, and sports processes of people with visual impairment. The individuals interviewed manifested the importance of technology to continue performing their personal and professional tasks and, in some cases, find entertainment sources or leisure time activities during confinement. For some of the people interviewed, prior use of technological tools has been a vital aspect to continue their educational processes: *from that change from the classroom to the virtual, a very important change was noted, I would use the computer for my homework but never imagined I would use it in such long schedules; I use JAWS, I have low vision and it was not too difficult to locate myself in the application we use in the university, I could locate myself easily and attended all the classes assigned and complied with all assignments [E3. Bucaramanga. Man]*.

Likewise, for the participants, using technologies in this type of situation has been identified as a facilitator of social inclusion, given that such permit conducting diverse activities of adapted manner and without any barriers: *technology is our helping hand because screen readers allow us to interact with mobile phones, computers and, well, today everyone has to do it like that because that is the work and study modality, anyway, so let’s say that facilitates things a lot [E1. Bogotá. Man].* Similarly, the technological skills of people with visual impairment are recognized as an aspect that can favor their labor inclusion, given the world’s mass migration to digital scenarios: *if someone with a disability today is looking for work and requires using technology they have won a part, thus, it is a large contribution* [*E1. Piedecuesta. Man*].

The computer literacy of some people with visual impairment has permitted maintaining physical activity as a fundamental aspect not only of the physical wellbeing but of social inclusion: *Generally, I train; now it is through Zoom [E5. Piedecuesta. Man]. Exercise continues, we are also working from home with the topic of the foundation, encouraging the kids at home to also keep practicing tennis for the blind [E9. Bogotá. Woman].* However, the rapid and generalized change of the daily dynamics that led to using on-line platforms due to the pandemic evidenced the digital gaps experienced by the country that are reflected in the different barriers for their use related with issues of not knowing the platforms, limited access to technologies, accessibility difficulties due to connectivity and economy for connection to an internet service: *sometimes, due to the very economic situations, of perhaps not having a mobile phone or access to internet [E3. Arauca. Woman].* In this same sense, not all people with visual impairment have access to applications or have prior training that allows them to confront adequately the challenge of the virtual realm: *A friend who is completely blind tells me that with the virtual classes I’ve not had access to any, because he also uses Jaws and with total blindness he has to adjust the computer commands and many do not locate him as he would like, so it became somewhat tedious to enter a class because the professor had to silence him when his microphone got activated and that was like treating him as special [E3. Piedecuesta. Man].*

Likewise, lack of direct contact with other people, professors and study peers, is an aspect missed from the educational experience and in the social experience: *I refer to sharing with my classmates to being in front of a professor, having that visual and physical contact, sitting behind a computer and looking at a screen always gets a bit tedious* *[E2. Bucaramanga. Man]*. *If we were face to face, you perceive this person, their odor is pleasant, the voice is heard differently, possibly some brush, this person can be delicate, has long or short hair, many more things that from the virtual you do not perceive [E1. Arauca. Woman].*

Technology has permitted developing initiatives of cooperation among people with visual impairment to somehow solve difficulties generated by isolation, through virtual scenarios some groups have generated calls to provide basic food to people who have been affected directly by the pandemic situation: *the food grocery campaign that began after the first week of the quarantine has not stopped; we delivered 140 to people that really nobody remembers or to people who get groceries at least once per month, then this campaign, has been done in Bogotá and Cundinamarca, with a form sheet that was made accessible. Minimum aid has arrived and people normally eat every day when they can, so these are very hard lives, and these are small actions [E9. Bogotá. Woman]. Through calls and E-mails, a little while ago we collected humanitarian aid to deliver groceries to those with a higher degree of vulnerability and, well, everybody approved and supported me [E3. Arauca. Woman].* These dynamics are recognized by those interviewed as an opportunity to enhance support ties among members from groups of people with disability: *Regarding the pandemic, there is something beautiful that says that resilience processes are fundamental and those support networks and creating bonds and, for me, that has been important [E4. Bogotá. Woman].*

## Discussion

Life experiences of people with visual impairment are unique and diverse, but also have in common that, to a greater or lesser extent, they need supplementary guarantees to live with full rights or participate under equal conditions with the rest of citizens in economic, social, and cultural life. (13) This study permitted understanding the changes in daily life emerging from the COVID-19 Pandemic in people with visual impairment from four cities in Colombia: Bogotá, Arauca, Bucaramanga, and Piedecuesta. In spite of the differences of context, no differentiating findings were identified where cultural or city issues could be identified as important aspects of the transformations. The situation of confinement has triggered a series of transformations in the dynamics of the individuals interviewed, identifying - among other things - how difficulties for mobility in autonomous and independent manner have increased due to the epidemiological measures and because of the hostile scenario experienced in the means of transport and in the interaction with others. People with visual impairment require, in some cases, support or orientation and they have run into very difficult panoramas in terms of the scant aid they find in the citizenry, given that they cannot get up close to request guidance or support due to the obligatory social distancing. These matters of mobility have been reported by the World Blind Union, which state that aspects, like using masks, not touching surfaces, not finding pedestrians willing to help, ignorance of guide dogs with respect to keeping physical distance, and changes regarding the level of noise in the streets originated additional challenges for orientation. (14)

According to the study on people with disability regarding COVID-19 in Latin America and the Caribbean, with the arrival of this health crisis and its devastating social and economic impacts, people with disability will be among the most affected, together with their families, which will worsen their situation of exclusion and marginalization. (15) In this study, testimonies from individuals interviewed account for the difficulties they are experiencing as a result of the poor opportunities they have to maintain their daily income or work contracts, given that the country still has a very large gap with relation to the effective labor inclusion of people with visual impairment, an issue that within the context of the pandemic has become even more evident. These matters had already been reported in a study by Moreno, which mentions that in Colombia although participation in the labor market is low for the total population in Colombia, this situation is even more precarious for people with who are visually limited, of which 38.1% work, 2.5% are looking for work, and 19.3% perform unpaid household chores. (16) These situations of precariousness explain, among others, the initiatives identified in this research in which people with disability have mobilized to provide basic food to families struck harder by the situation.

Moreover, the context of the pandemic has evidenced the need to strengthen processes of accessibility to information and of computer literacy of people with visual impairment as a mechanism to favor their educational and labor inclusion. During the development of this research, it was possible to identify that technological tools become facilitators in the individuals interviewed for the continuity of their daily activities; however, it is important to reflect on the technological gap that persists in the country, given that in some cases those interviewed did not have the minimum materials to guarantee their connectivity, like mobile phones, computers, or connection to the internet or with prior formation processes that permit their adequately manipulating the devices. This issue has been reported in another study, which shows that digital gaps exist in access to computers, the internet and skills in the use of these devices and virtual platforms by people with disability. The impacts of COVID-19 will broaden disparities for these individuals in access to virtual learning through the use of technological media, hence, it is necessary to prioritize reasonable adjustments, like modifications or adaptations to these digital environments in function of the diverse social peculiarity of these individuals toward quality and inclusive education. (17)

For the specific case of education, the situation experienced as a consequence of COVID-19 has obligated adapting the educational system, designed for classroom teaching, to a “remote” modality. Within this context, it becomes more important to advance toward the creation of different guidelines of the universal design of learning that favors the teaching-learning process and, consequently, permits students with visual impairment to exert their right of access to an educational environment designed to help them reach their maximum potential. (11) Finally, the epidemiological measures to contain the virus have affected the autonomy and independence of those interviewed, which was already reported by other organizations in which people describe repeatedly feelings of frustration, anxiety, anger, low self-esteem, and discouragement caused by the loss of autonomy and independence, without having the same access and opportunities as the rest. (14)

All societies in the world are in a learning process that has implied assuming new practices for self-care and collective care of health. Restrictions that obligate staying at home that do not keep in mind the needs of people with disability create disturbances and new risks for their autonomy, health, and lives. As already reported, COVID-19 threatens with exacerbating these disparities, particularly in low- and middle-income countries. (9) Due to this, the country is obligated to offer guarantees for the measures taken during the pandemic to not affect disproportionately people with visual impairment and attempt against the compliance of their rights. It is necessary to establish clear intervention mechanisms that permit the effects and serious impacts due to COVID-19 to be minimized. (10)

This work permitted understanding changes in daily life emerging from the COVID-19 pandemic in people with visual impairment from four cities in Colombia: Bogotá, Arauca, Bucaramanga, and Piedecuesta. People with visual impairment participating in this study report transformations related with barriers for their mobility, as well as for using public transportation, which is a critical aspect that can affect maintenance of their autonomy and independence. Another key aspect identified in the research has to do with using technological tools as facilitators for the continuity of their educational and work activities; however, this is an aspect that was not homogeneous in all the participants, given that some of them had no computer literacy required or the basic inputs for connectivity. They also state difficulties in continuing their work activities and maintain wages. Even within the universality of the pandemic, all our experiences and individual contexts are unique, thereby, it is important to propose the need to initiate inclusive approaches when planning responses to the pandemic and post-pandemic management. It is necessary, among other things, to advocate for the development of an effective implementation of accessibility standards with principles of universal development to favor the right to education and work of people with visual impairment. Likewise, establishing articulation points with educational institutions to monitor the accessibility conditions of students and, thus, take measures that permit the permanence of the student body and thus avoid their desertion.

With the purpose of identifying who are the people with visual impairment most affected in the country, especially those who experience multiple interweaved forms of discrimination, it is necessary to promote the collection of data that includes in their research, information broken down about people with visual impairment, which will permit prioritizing intervention actions during the post-pandemic phase. Finally, the current situation may be seen as an opportunity for decision makers to establish a reform on how care has been provided to people with visual impairment, thus, it is necessary to work closely with representatives from organizations of people with disability to identify priority lines of post-pandemic work, given that it is likely that the challenges will be greater.
